# Response of Leptomeningeal Metastases in *EGFR*-Mutated Non-Small-Cell Lung Cancer to Afatinib in the Absence of Radiotherapy

**DOI:** 10.1155/2019/1939703

**Published:** 2019-09-16

**Authors:** Néstor Llinás-Quintero, David González-Hoyos, Andrés Yepes, Diego A. Herrera, Sebastián Peláez-Arroyave, Carlos Caicedo-Zamudio, Erick Blanco-Daza, Javier Cuello-López

**Affiliations:** ^1^Clinical Oncology Group, Fundación Colombiana de Cancerología-Clínica Vida, Medellín, Colombia; ^2^School of Medicine, CES University, Medellín, Colombia; ^3^Diagnostic Imaging Group, CEDIMED, Medellín, Colombia; ^4^School of Medicine, UPB University, Medellín, Colombia

## Abstract

Palliative radiotherapy is currently the medical standard of care for non-small-cell lung cancer (NSCLC) patients with symptomatic CNS and leptomeningeal disease. We report the case of a 62-year-old male patient with EGFR *mutation* (*del*19+) NSLC with symptomatic lymph node, bone, CNS, and leptomeningeal metastases. Taking into account on one hand the response to tyrosine kinase inhibitors (TKIs) and on the other hand the short- to medium-term side effects of radiotherapy and the lack of timely availability in our healthcare system, the patient was treated with afatinib (40 mg daily) and exhibited a rapid response with improvement of neurological symptoms. The patient presented partial response of extracranial, CNS, and leptomeningeal lesions at 3, 6, and 12 months of treatment, currently completing 16 months of progression-free survival despite presenting mild dermatological and gastrointestinal toxicities. Afatinib is an effective and safe option in patients with NSLC EGFR *mutation del*19+ with CNS and leptomeningeal compromise avoiding or delaying radiotherapy and its side effects, especially in countries where there is a lack of access to this kind of therapy.

## 1. Introduction

Lung cancer is the leading cause of cancer death worldwide [1, 2]. In these patients, central nervous system (CNS) metastases are estimated to occur in 30 to 50% of patients, which carries a poor prognosis of 4.7% in a 5-year relative survival rate, which is even poorer if there is symptomatic leptomeningeal disease [1–3].

Among EGFR-mutant NSCLC patients, leptomeningeal disease occurs in approximately 9% of cases, with a median overall survival of 3 months [4–8]. Thus, treatment goals are improved quality of life and prolonged survival. To date, studies providing evidence on how to best approach these patients are limited; nonetheless, currently used treatment regimens include radiotherapy, intrathecal chemotherapy, and systemic therapy, which—despite achieving various outcomes—carry a poor prognosis [5–7, 9–11].

Treatment of brain metastasis involves whole brain radiation therapy (WBRT), stereotactic radiosurgery (SRS), and surgery. However, with the improvement of systemic therapy, especially for treatment of tumors presenting anaplastic lymphoma kinase (ALK) translocations and epidermal growth factor receptor (EGFR) mutations, cancer control can be achieved. For instance, the third-generation EGFR tyrosine kinase inhibitor (EGFR-TKI) osimertinib has been evaluated in prospective trials conducted in patients with brain metastases and showed significant improvement in progression-free-survival (PFS) compared to first-generation EGFR-TKI. However, the cost of treatment has limited the access to this therapy [3, 5, 6, 11–20]. Furthermore, in patients with advanced EGFR-mutant NSCLC, afatinib—a second-generation EGFR-TKI—has demonstrated activity against CNS and leptomeningeal metastases (LM) and to perform superiorly to gefitinib—a first-generation EGFR-TKI [21–24].

## 2. Case Description

A 62-year-old male with past medical history of hypertension, type 2 diabetes, and fibromyalgia presented to our clinic with complaints of back pain and dyspnea. The chest X-ray showed bilateral micronodular opacities and a nodule in the paramediastinal and aortic regions. Due to worsening of symptoms, a CT scan was performed, and a 35 × 27 × 33 mm speculated mass in the right upper lobe, bilateral mediastinal lymphadenopathies, and bilateral apical diffuse reticulonodular and micronodular opacities were identified. On bronchoscopy, no endobronchial lesions were identified, with a high suspicion of malignancy in aspiration cytology. PET-CT was performed and showed bilateral diffuse pulmonary metastatic disease, lesions in most vertebrae (C2, C6, C7, T2, T4-T6, T8-T12, L1-L5, sacral segments S1 and S2, and both iliac spines), and mediastinal lymph node involvement. A videothoracoscopy-guided segmental lobectomy plus mediastinal lymphadenectomy was performed for pathology confirmation. The pathology report indicated moderately differentiated lung multifocal lepidic-predominant adenocarcinoma, with lymph node involvement and with visceral, pleural, lymphovascular, and border invasion.

The patient was referred to clinical oncology, as he continued presenting symptoms complaining of persistent cough, weight loss, headache, and dyspnea, presenting a NYHA functional class III/IV and poor performance status (Karnofsky Performance Status Scale 70%). Upon physical examination, bibasilar rales and a positive percussion test in the dorsal vertebrae were identified. Laboratory tests showed normal renal, hepatic, and hematologic function. The patient was diagnosed with stage IVB (T2N3M1c) NSCLC adenocarcinoma with bone, mediastinal lymph node, and contralateral lung involvement. A brain MRI showed multiple nodular lesions in the brain and cerebellar parenchyma with leptomeningeal enhancement. A spinal MRI confirmed the metastatic involvement in the vertebrae, as indicated by the previous PET-CT scan, but without epidural infiltration. In addition, positive *EGFR* exon 19 deletion was also identified.

**Figure 1:**
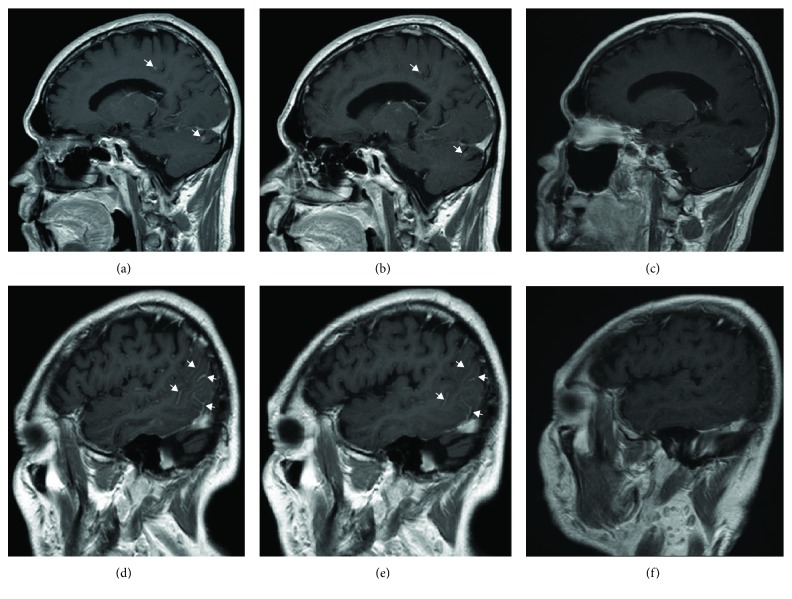
T1 WI postgadobutrol administration sagittal slices. (a, d) Nodular enhancing lesions between 1 and 4 mm (arrows) at the centrum semiovale and left cerebellar hemisphere. Additional leptomeningeal enhancement was noticed at the pial surface of the left posterior quadrant (arrows). (b, e) Images obtained 7 months later. There is evident reduction in lesion size, and fewer leptomeningeal enhancement is noticed. (c, f) Images obtained 11 months from first study; no lesions or enhancement noted.

**Figure 2:**
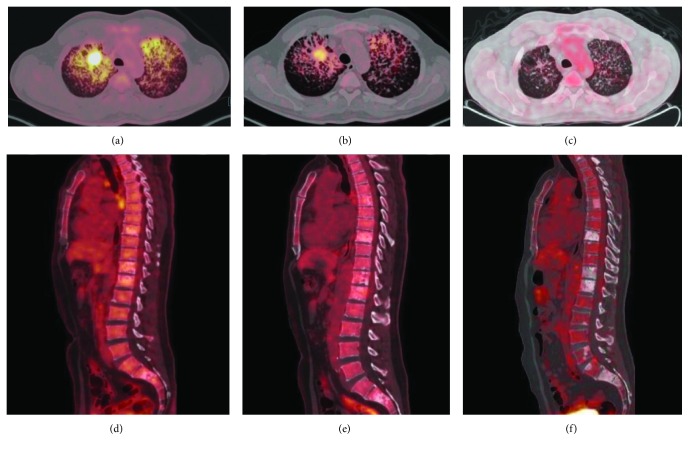
18-FDG PET CT fusion images: axial and sagittal slices. (a, d) Right upper lobe mass with abnormally increased metabolism with surrounding lymphangitic carcinomatosis on both upper lobes with increased metabolic activity as well. On sagittal slices, multiple mixed type (blastic/lytic) metastatic lesions affecting axial skeleton. (b, e) Images obtained 7 months later showed marked decrease on metabolic activity on both pulmonary and skeletal lesions. (c, f) Images obtained 11 months from first study demonstrate the absence of metabolic activity on both primary site and spinal lesion.

Due to the lack of timely access to radiotherapy, and considering its associated side effects, radiation therapy was postponed, and palliative care was initiated with treatment with a tyrosine kinase inhibitor, because of his *EGFR* mutational status. The patient was started on a 40 mg/day dose of afatinib treatment, administered orally, until there was evidence of disease progression or drug tolerance. Additionally, the patient received antiresorptive therapy with intravenous bisphosphonates every month to treat his bone metastasis. Immediately after initiating therapy, the patient exhibited rapid symptom and performance status improvement and was able to tolerate treatment; however, he presented grade 1 gastrointestinal toxicity and grade 2 dermatological toxicity. Follow-up images at 3, 6, and 12 months of treatment showed partial response of extracranial, CNS, and leptomeningeal lesions (Figures 1 and 2). To date, the patient has completed 16 months of treatment, displaying adequate tolerance and partial response to therapy, as well as a preserved quality of life and no evidence of disease progression.

## 3. Discussion

*EGFR* mutations are observed in 15% of lung cancer cases and have been associated with metastatic tropism to the brain, as well as to predict sensitivity to tyrosine kinase inhibitors (TKIs) [3, 5, 7, 8, 13, 25]. A retrospective study found a frequency of 7.8% of the lepidic subtypes with 68.8% of them with EGFR mutation [26]. In non-small-cell lung cancer, TKIs have proven efficacy in improvement of progression-free survival, response rate, and quality of life when compared to chemotherapy in patients with an *EGFR* mutation and are included in the current standard of care for patients with symptomatic brain metastasis together with WBRT [3–6, 13, 25–28].

The incidence of leptomeningeal carcinomatosis (LC) in *EGFR*-mutant NSCLC patients is 9%, and while median overall survival (OS) is 3-4.5 months, 44% have prolonged survival of more than 6 months. In a retrospective analysis of patients with LC by NSCLC with *EGFR* mutations, TKI therapy after LC diagnosis was an independent predictive factor of extended survival (median OS 10.0 vs. 3.3 months, HR = 0.218, *p* < 0.001), whereas poor Eastern Cooperative Oncology Group performance status (*p* < 0.001, HR = 3.657) was a predictor of poor survival [29, 30]. In that study, the active treatment with WBRT did not prolong OS for EGFR-mutated patients.

WBRT has been used as a therapeutic strategy in patients with brain and leptomeningeal metastases to relieve symptoms, to reduce bulky or nodular disease, and to correct CSF flow [31]. However, given its short and long-term adverse effects, and the increasing survival with the use of TKIs, even in patients with cerebral involvement, WBRT is being used less in patients with NSCLC with oncodriver gene mutations [6, 27]. Recently, Wang et al. published a meta-analysis that evaluated the role of WBRT as a treatment associated with TKIs in patients with NSCLC with brain metastases and EGFR mutation [10]. This meta-analysis included seven eligible studies for a total of 1086 patients. Compared to TKI alone, upfront WBRT plus TKI showed better PFS (HR = 0.72, 95% CI: 0.53-0.97, *p* = 0.028) and OS (HR = 0.70, 95% CI: 0.53-0.93, *p* = 0.015). Metaregression analyses and subgroup analyses showed that while patients with a limited number of brain metastases (<3) benefited from WBRT as evidenced by improved OS (HR = 0.54, 95% CI: 0.41-0.72, *p* ≤ 0.001), patients with more than 3 brain metastases did not show OS benefit by undergoing WBRT [10].

Emerging evidence indicates that second- (afatinib) and third-generation (osimertinib) EGFR TKIs effectively penetrate the blood-brain barrier (BBB) and therefore represent viable treatment options for CNS lesions and can reduce the risk of CNS progression. These agents should be therefore considered as first-line treatment options in patients with *EGFR* mutation-positive NSCLC who have brain metastases and/or LM [7, 8, 32].

Evidence of the activity of afatinib against brain metastases has also been demonstrated by recent risk analyses of the LUX-Lung 3 and 6 studies [24]. In patients with a target brain lesion at the start of afatinib treatment, the risk of CNS progression (34%) was lower than the risk of non-CNS progression (48%). *De novo* CNS progression was observed in only 5% vs. 71% in non-CNS progression of patients after 24 months [13]. A combined analysis of the LUX-Lung 3 and LUX-Lung 6 trials showed benefit in PFS with afatinib as first-line treatment compared with chemotherapy in patients with asymptomatic CNS metastases (8.2 vs. 5.4 months; hazard ratio, 0.50; *p* = 0.0297) [24].

Osimertinib, a third-generation TKI that selectively inhibits both *EGFR*-TKI-sensitizing and *EGFR* T790M resistance mutations, has been approved as first-line treatment for *EGFR* mutation-positive NSCLC and as second-line after *EGFR*-TKI treatment in patients with *EGFR* T790M resistance mutations [16]. Osimertinib has a high bioavailability in the CNS [18]. In the FLAURA study, 556 patients with NSCLC and positive *EGFR* mutation were randomly assigned to osimertinib or standard of care (erlotinib or gefitinib). In this trial, there was a significantly longer PFS in the osimertinib group (18.9 versus 10.2 months; hazard ratio 0.46, 95% CI: 0.37-0.57) [18]. A subanalysis of the FLAURA study in patients with CNS involvement included 128 patients of the 200 that had available brain scans at the baseline. This subanalysis showed that median CNS progression-free survival was not reached with osimertinib (95% CI, 16.5 months to not calculable) and was 13.9 months with standard EGFR-TKIs (95% CI, 8.3 months to not calculable) (hazard ratio, 0.48; 95% CI: 0.26 to 0.86; *p* = 0.014) [19].

In our case, we were unable to provide treatment with osimertinib since it is not currently approved in our country; thus, despite evidence of brain and leptomeningeal metastases, the patient was started on treatment with afatinib, taking into account the evidence on clinical response of *EGFR*-mutated NSCLC without prior radiotherapy. This therapeutic strategy allowed postponing radiotherapy and its side effects. In addition, in some cases, access to radiotherapy can be difficult and delayed, putting the patient's life at risk. The partial response to treatment with improvement of his performance status and an outstanding progression-free survival strongly supports the safety and effectiveness of this therapy. Afatinib is an effective and relatively safe option in patients with *EGFR* mutation *del*19+ with symptomatic CNS and leptomeningeal metastases, especially in countries where the access to radiotherapy is still limited. Additional studies should be conducted to obtain more information on the safety and efficacy of tyrosine kinase inhibitors, especially in patients with CNS and leptomeningeal metastatic involvement.
